# Prevalence of venous thromboembolism risk factors in hospitalized patients at the University of Nigeria Teaching Hospital, Enugu Nigeria

**DOI:** 10.4314/ahs.v22i2.83

**Published:** 2022-06

**Authors:** Eyiuche Doris Ezigbo, Theresa Ukamaka Nwagha, Victor Chinedu Nwuzor

**Affiliations:** 1 Department of Medical Laboratory Sciences, University of Nigeria Enugu Campus, Enugu, Nigeria; 2 Department of Haematology and Immunology, University of Nigeria Teaching Hospital, Enugu, Nigeria

**Keywords:** Venous Thromboembolism, Risk factors, D-dimer, Patients, Nigeria

## Abstract

**Background:**

Venous thromboembolism (VTE) is a common and lethal disease. Assessing the risk factors will help to modify exposures.

**Aim:**

This study, assessed VTE risk factors in hospitalized patients at the University of Nigeria Teaching Hospital, Enugu.

**Patients and Methods:**

This was an observational, case-control study. Three hundred and fifty (350) patients were recruited for the study: This comprised 150 medical patients, 140 surgical patients and a population of 60 healthy control group. Subjects were evaluated once using the Caprini risk assessment model (RAM).

**Results:**

Over 50% of all hospitalized patients, were at risk for VTE. Surgical patients were at a higher risk than medical patients. Hemoglobin concentration was associated with the risk of VTE in surgical patients, while d-dimer was associated with VTE risk in medical patients.

**Conclusion:**

This study shows a high prevalence of VTE risk factors among hospitalized patients at the University of Nigeria Teaching Hospital.

## Introduction

Venous thromboembolism describes the development of blood clumps in the vein, which is, involvement of erythrocytes, fibrin, platelets, and leucocytes in the formation of a clump inside a vein. Depending on where the clot forms; if on a deep vein, of the leg, it is termed deep vein thrombosis (DVT). If the clump breakup and moves to the lungs to settle, it is called a pulmonary embolism (PE), (VTE=DVT + PE). Globally, VTE is one of the leading cause of hospital death; it is the third most common cause of cardiovascular diseases globally^1^ and up to 60% of cases of VTE are hospital-associated. VTE occurrence in whites of European origin is above 1 per 1000; the incidence among descents of African and Asian origin could be higher and lower, respectively^2^,^3^. In sub-Sahara, Africa VTE is a huge challenge, 1 out of 2 in-patients are at risk of VTE, and 43.8% of patients in the surgery wards are at risk of VTE, while 62.3% of the patients in the medical ward are at risk of VTE 4.

Some of the hospital-associated Risk factors include surgery-related risk factors such as abdominal surgery, pelvic and orthopedic surgery, major trauma or burns,/ indwelling catheters. Medically related risk factors: cancer, pregnancy and post-partum, congestive heart failure, myocardial infarction, stroke, obesity, other VTE associated risk factors include: oral contraceptive use, chemotherapy, increasing age, varicose veins, smoking 5.

Management of VTE includes the use of mechanical methods such as the use of (a) Anti-embolism stockings (AES)^6^. (b) Periodical pneumatic compression, (c) Foot impulse devices or foot pumps raises venous outlet and decrease stagnancy in immobilized patients. Pharmacological prevention involves perioperative administration of anticoagulant drugs for example: Unfractionated heparin, low-molecular-weight heparins (LMWHs), oral Direct Factor Xa inhibitors, etc.

Heit et al in 2006 7 proposed the incidence of VTE to be high in Africans 7, the prevalence of VTE in Africa was also reported to be high following surgery, pregnancy, and post-partum 8, In risk factor-adjusted models, blacks had a higher rate of VTE than whites^2^. Despite these assumptions, there are limited data on the prevalence of VTE and its associated risk factors in Nigeria. Recently, in a study on post-mortem data 9, there was a prevalence of 2.9% in VTE with a male to female ratio of 2.6%. Interestingly, in another study that looked at the knowledge and practice of VTE risk and prophylaxis, only about 33.3% of surgeons have good knowledge about DVT prophylaxis in some hospitals in Nigeria^10^. Notwithstanding, 90.5% of these surgeons have encountered DVT and 83.5% of them have encountered PE in their practice. Identifying of patients at risk of VTE before or on admission in the hospital, or in the outpatient setting, is very important in starting necessary preventative management or treatment.

## Patients and Methods

### Study design and population

This was an observational, case-control study on the prevalence of VTE risk factors in hospital patients at UNTH. Subjects were evaluated once using the Caprini risk assessment model (RAM) 11. It contains the risk factors to developing VTE in their order of increasing importance in boxes that were assigned points. Patients were recruited from the surgical and medical wards of UNTH. Patients were eligible for the study if they have been on the hospital bed for not less than 72hours. The semi-ambulatory and completely non-ambulatory ones were selected for the study. The Medical doctor in collaboration with the research team administered the risk assessment questionnaire.

Three hundred and fifty (350) patients patents were recruited for the study: this comprised 150 medical patients, 140 surgical patients, and a population of 60 healthy subjects were enrolled as the control group. In the medical group, patients who were admitted for treatment of a medical illness and /or were admitted for a major traumatic event that requires no major operation were included in the study. In the surgical group, patients who have undergone surgical operations were included in the study. Patients were excluded if they were not able to give consent for the study. The updated 2013 Caprini risk assessment tool for venous thrombosis risk factor, served as the questionnaire for the study. It contains the risk factors to developing VTE in their order of increasing importance in boxes that were assigned points. Written informed consents were signed by all subjects and the University of Nigeria Research Ethics Committee approved the study,(NHREC/05/01/2008BFWA00002458-1RB00002323

### Blood sampling and laboratory analysis

6.5ml of blood was obtained from each subject by clean venipuncture and under aseptic conditions. 2ml of this blood was dispensed into a plastic tube containing 1.0mgm/l ethylene-diamine tetra acetate (EDTA) for complete blood count and ABO RhD Blood grouping. The remaining aliquot of 4.5ml of blood was dispensed into 500ul of 3.2% trisodium citrate specimen bottle for the determination of the D-dimer level.

### Platelet – poor plasma preparation

4.5ml of blood in 3.2% trisodium citrate was spurn 1hr after collection at 3000g for 10 mins and plasma extracted, liquated, and stored -80 until analysis.

Complete blood count (CBC): the BC 5300 auto hematology analyzer Mindry China was used for the CBC according to the manufactures instructions.

D-dimer measurement: Normal d-dimer level is important in ruling out the diagnosis of evolutive DVT or PE=VTE. We, therefore, assessed the d-dimer levels as part of the investigations of patients for VTE risk assessment. The d-dimer ELIZA assay kit from Technoclone Austria was used according to the manufacture's instructions. A negative test was defined as a d-dimer value < 500ngmL.

### Statistical analysis

#### Frequency and percentages were used to indicate the distribution of categorical parameters

For comparison of two groups, Mann Whitney U test was used, for more than two groups the Kruskal Wallis H test was used. The values were expressed as median and the interquartile range (25^th^ and 75^th^ percentiles). Binary logistic regression was used to determine the associations of blood count parameters and d-dimer with a high risk of VTE. Both univariate and multivariate analysis adjusting for socio-demographic variables were carried out.

P-value of < 0.05 was considered significant. The statistical analysis was carried out using the Statistical Package for Social Sciences (IBM SPSS Statistics), version 23.0. Armonk, NY.

## Results

Overall, 350 subjects were enrolled into the study. Detailed information about the study population, which includes medical patients, surgical patients and apparently healthy control subjects, are presented in Table 1. The median and the interquartile range (25^th^ and 75^th^ percentiles) of the quantitative variables that include age, weight, height, body mass index (BMI) and the laboratory parameters are presented in the table. The frequency and percentage of gender and venous thromboembolism (VTE) risk factor assessment are also presented in the table.

**Table 1:** Characteristics of study population

Characteristics	Medical patients (n=150)	Surgical patients (n=140)	Control subjects (n=60)
**Age (years) Median (IQR)**	47 (20–71)	47 (16–76)	22 (19–24)
**Gender**			
**Male n (%)**	75 (50%)	94 (67.1%)	18 (30%)
**Female n (%)**	75 (50%)	46 (32.9%)	42 (70%)
**Weight (kg) Median** **(IQR)**	66.5 (60.0 – 69.0)	58.5 (52 – 68)	62 (57 – 68)
**Height (m^2^) Median (IQR)**	1.51 (1.37 – 1.61)	1.47 (1.41 – 1.57)	1.55 (1.42 – 1.61)
**BMI (kg/m^2^) Median** **(IQR)**	28.8 (26.0 – 32.9)	28.6 (25.4 – 30.7)	28.0 (23.6 – 30.8)
**VTE Risk factor** **assessment**			
**Low risk (Score = 0–2)**	20 (13.3%)	-	-
**Moderate risk** **(Score=3–4)**	52 (34.7%)	38 (27.1%)	
**High risk (Score ≥5)**	78 (52.0%)	102 (72.9%)	-
**Laboratory** **parameters**	Median (IQR)		
**WBC (×10^9^/L)**	6.6 (4.5 – 8.1)	5.4 (4.2 – 7.3)	4.6 (4.1 – 5.7)
**HGB (g/dl)**	11.5 (9.2 – 12.5)	12.8 (11.0 – 13.9)	12.2 (11.1 – 13.0)
**PLT (×10^9^/L)**	259.0 (192 –326)	220.0 (165 –261)	211.5 (171 –265)
**MPV (fl)**	8.2 (7.5 – 8.7)	7.6 (7.4 – 8.7)	8.7 (8.4 – 9. 3)
**PCT (%)**	0.20 (0.18 – 0.26)	0.18 (0.14 – 0.22)	0.19 (0.15 – 0.23)
**PDW (fl)**	13.3 (12.1 – 14.9)	12.9 (11.2 – 14.2)	14.5 (14.0 – 15.2)
**PLCR (%)**	13.1 (10.0 – 15.6)	10.4 (7.0 – 18.0)	16.8 (13.9 – 20.5)
**D-dimer (ng/ml)**	244.6	375.3	116.6 (79.2 – 158.4)
	(117.0 – 557.2)	(129.8 – 833.5)	

Result reported as Frequency and percentage for qualitative data, Median (IQR - Interquartile range) for quantitative data BMI - Body Mass Index, VTE - Venous thromboembolism, HGB - Haemoglobin, WBC - White blood cell, PLT - Platelet, MPV - Mean platelet volume, PCT - Plateletcrit, PDW - Platelet volume distribution width, PLCR - Platelet larger cell ratio

**Figure 1:**
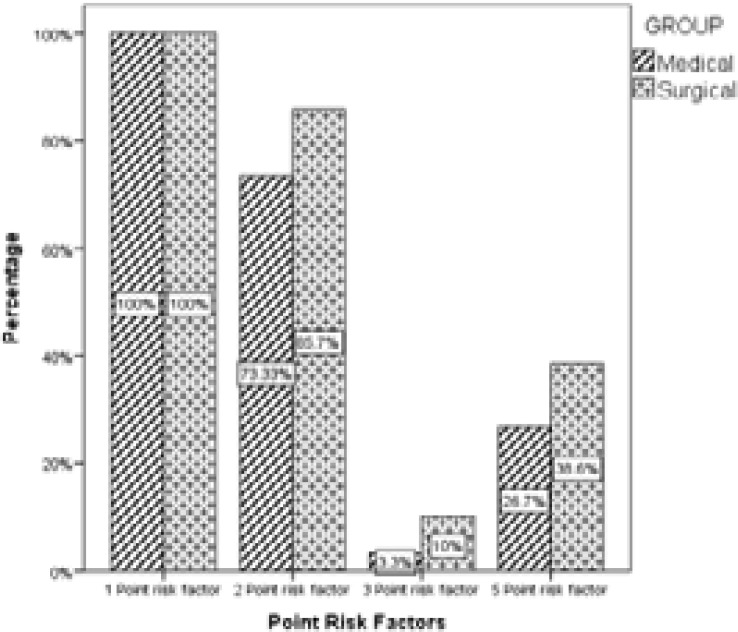
Prevalence of VTE risk factors in hospitalized patients

**Figure 2:**
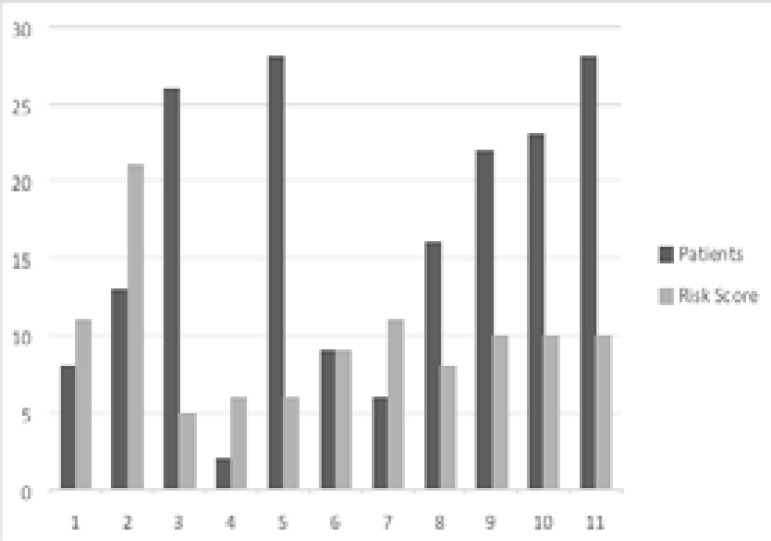
Patients with high-risk scores

Figure 1 shows the prevalence of VTE risk factors among hospitalized patients. Of the 93.1% (270/290) of the patients with VTE risk score ≥3, 130/150(86.7%) are medical patients, while 140/140(100%) is the surgical patients. Figure 2 shows a graphical representation of the patients with high-risk scores. The set point for the highest risk score in this patient population is 10.

The association of the ABO blood group with VTE risk was also analyzed, the ABO blood group was not statistically associated with VTE risk factor either in the medical patients, Fisher exact test -5.84, p=0.134 or in the surgical patient, fisher exact test -1.490, p=0.604.

In the table, 3 and 4 binary regression was used to determine the association of blood count parameters and d-dimer in predicting the risk of VTE. Multivariate analyses, adjusting for socio-demographic variables (sex, age, and BMI), as additional covariates with blood count parameters and d-dimer were further carried out to ascertain if the associations persist.

The relationships between the laboratory parameters seen at each Caprini RAM score (CRS) above 5 in the study populations are presented in tables 5 and 6. For the medical patients table 5, seventy-eight (78) out of the 150 medical patients had CRS greater than 5. Table 6 shows the correlations between the laboratory parameters seen at each CRS score above 5 in surgical patients. Out of the 140 surgical patients, 102 of them had CRS above 5.

**Table 5:** Correlations between the laboratory parameters seen at each Caprini RAM score above 5 in medical patients (n=78)

Parameters		WBC	RBC	PLT	MPV	PCT	PDW	PLCR
**RBC**	r	-0.468						
	P-value	<0.001*						
**PLT**	r	-0.176	-0.015					
	P-value	0.123	0.897					
**MPV**	r	-0.524	0.266	0.008				
	P-value	<0.001**	0.018*	0.941				
**PCT**	r	-0.182	-0.011	0.966	0.114			
	P-value	0.110	0.923	<0.001*	0.318			
**PDW**	r	0.217	-0.069	0.497	-0.074	0.600		
	P-value	0.056	0.549	<0.001**	0.521	<0.001**		
**PLCR**	r	0.005	-0.108	-0.259	0.386	-0.077	0.390	
	P-value	0.963	0.344	0.022*	<0.001**	0.501	<0.001**	
**D**	r	-0.234	-0.104	-0.167	0.126	-0.184	-0.067	-0.036
**Dimer**	P-value	0.039*	0.365	0.144	0.273	0.107	0.557	0.756

r - Pearson correlation coefficient

* Correlation significant at p<0.05

** Correlation significant at p<0.01

WBC - White blood cell, RBC - Red blood cell, HGB - Haemoglobin, HCT - Haematocrit, MCV - Mean cell volume, MCH - Mean cell haemoglobin, MCHC - Mean cell haemoglobin concentration, PLT - Platelet, MPV - Mean platelet volume, PCT - Plateletcrit, PDW - Platelet volume distribution width, PLCR - Platelet larger cell ratio

**Table 6:** Correlations between the laboratory parameters seen at each Caprini RAM score above 5 in surgical patients (n=102)

Parameters		WBC	RBC	PLT	MPV	PCT	PDW	PLCR
**RBC**	r	-0.402						
	P-value	<0.001**						
**PLT**	r	0.211	-0.407					
	P-value	0.034*	<0.001**					
**MPV**	r	-0.435	0.337	-0.500				
	P-value	<0.001**	0.001**	<0.001**				
**PCT**	r	0.081	-0.280	0.960	-0.313			
	P-value	0.421	0.004**	<0.001**	0.001**			
**PDW**	r	-0.376	0.228	0.073	0.494	0.261		
	P-value	<0.001**	0.021*	0.468	<0.001**	0.008**		
**PLCR**	r	-0.312	0.270	-0.590	0.938	-0.433	0.325	
	P-value	0.001**	0.006**	<0.001**	<0.001**	0.000**	0.001**	
**D Dimer**	r	0.360	0.225	-0.367	0.217	-0.377	-0.087	0.249
	P-value	<0.001**	0.023*	<0.001**	0.029*	<0.001**	0.386	0.012*

r - Pearson correlation coefficient

* Correlation significant at p<0.05

** Correlation significant at p<0.01

WBC - White blood cell, RBC - Red blood cell, HGB - Haemoglobin, HCT - Haematocrit, MCV - Mean cell volume, MCH - Mean cell haemoglobin, MCHC - Mean cell haemoglobin concentration, PLT - Platelet, MPV - Mean platelet volume, PCT - Plateletcrit, PDW - Platelet volume distribution width, PLCR - Platelet larger cell ratio

## Discussions and Conclusion

Here, we studied the prevalence of VTE risk factors in medical and surgical patients and studied the relationship of complete blood count indices and d-dimer with the risk of VTE. The VTE risk factors identified in this study include: (5points risk factors) hip, pelvis or leg fracture, stroke, multiple traumas, acute spinal cord injury. (2 points risk factors), malignancy, major surgery, patient confined to bed, immobilizing plaster cast. (1 point risk factors); minor surgery planned, history of prior major surgery, varicose veins, history of inflammatory bowel disease, swollen legs(current), obesity (BMI>25), acute myocardial infarction, congestive heart failure(< 1 month), sepsis(<1 month), lung disease(pneumonia), abnormal pulmonary function, medical patient currently at bed rest, oral contraceptives or hormone replacement therapy (HRT). The highest CRS seen in this study was 21in medical patients and 20 in surgical patients. Patients with a CRS of 10 and above were 37 out of 150 medical patients (24.7%) and 54 out of 140 surgical patients (38.6%).

The major outcomes of this study were that the percentage of surgical patients with high VTE risk factors was greater. The WBC, platelet count, PCT, and d-dimer levels were associated with the risk of VTE in medical patients after correction for sex, age, and BMI. In the surgical group increased WBC, hemoglobin concentration was associated with the risk of VTE after adjustments.

First, we examined the mean difference in the hematological profile of the study population and observed a statistically significant difference between the groups. We also examined the associations of full blood count (FBC) indices with the risk of VTE in medical patients and surgical patients. Leukocytes, mean platelet volume, and red cell distribution width has been reported as biomarkers for thrombosis in subjects with and without cancer. Besides, we examined the d-dimer levels of our study population.

Our findings regarding the prevalence of VTE risk factors in hospital patients at the University of Nigeria Teaching Hospital did not agree with earlier studies. The prevalence of VTE risk factors was reported to be higher in medical patients than in surgical patients^4^. In this research, we observed a high prevalence of VTE risk factors in surgical patients. This may be given the explanation that patients in this study were not on any VTE prophylaxis during the time of the study.

Our results regarding the full blood count (FBC) parameters are partially in agreement with previous studies. The red blood cells (RBCs) are significantly involved in normal hemostasis and pathologic thrombosis. High hematocrit and hemoglobin have been related with long term risk of VTE in the general population^12^,^13^,^14^,^15^. In cancer patients, RDW and RBC parameters were not independently associated with the risk of VTE 16. Preoperative infusions of red blood cells may significantly relate to the development of new VTE or progression 17. In univariable and multivariable analysis, we observed an association of hemoglobin concentration with the risk of VTE in surgical patients. Furthermore, total white blood count was associated with the risk of VTE in medical patients in multivariable analysis, while in surgical patients the association of total WBC and VTE risk was significant at invariable and multivariable analysis. Other studies have reported a high monocyte count to be associated with past VTE^18^. Platelets play a key role in thrombosis^19^,^20^. Interestingly, high platelet count was found to be associated with the risk of VTE in medical patients after multivariable analysis, while in surgical patients it was only significantly associated with VTE risk after univariable analysis. The association of high platelet count with VTE risk was reported in cancer patients^21^.

D-dimer is generally used to exclude VTE in Europe and North America; it is a global indicator of coagulation activation and fibrinolysis. D-dimer level (>70th percentile of controls) is independently associated with a 2.2-fold higher risk for first venous thrombosis 22. It also relates positively to the occurrence of a future first thrombosis in a population-based cohort study^23^. In Nigeria it has been reported as a marker for deep vein thrombosis in cervical cancer patients^24^, in Japan, high plasma d-dimer level was reported to be an independent risk factor for postoperative VTE^25^. In our study, we observed that d-dimer was associated with the risk of VTE in medical patients only. Its usefulness in surgical patients may require a wide study.

This study shows an increased occurrence of VTE risk among hospitalized patients at the University of Nigeria Teaching Hospital, Enugu. More than half (50%) of all hospitalized patients were at risk for VTE. Patients at the surgical wards were at greater risk of VTE than those at the medical wards. Patients were not on any antithrombotic regimen during the time of this study. The reason may be multifactorial ranging from lack of information on available types of prophylaxis, dosage, or fear of hemorrhage^26^. Studies that will validate VTE events in these at-risk patients will add significantly to the management of these patients.

Future studies should update patient VTE scores as additional problems may occur during hospitalization that might change the score, and hence necessitating prophylaxis in cases of low initial score. We also suggest that future studies should include data on thrombotic events.

## Figures and Tables

**Table 2:** Haematological profile and D-dimer levels of the study population

Parameters	Medical patients (n=150)	Surgical patients (n=140)	Control subjects (n=60)	P-value
**WBC (×10^9^/L)**	6.6 (4.5 – 8.1)^a^,^b^	5.4 (4.2 – 7.3)^a^	4.6 (4.1 – 5.7)	<0.001*
**HGB (g/dl)**	11.5 (9.2 – 12.5)^a^,^b^	12.8 (11.0 – 13.9)	12.2 (11.1 – 13.0)	<0.001*
**PLT (×10^9^/L)**	259.0 (192 -326)^a^,^b^	220.0 (165 -261)	211.5 (171 -265)	<0.001*
**MPV (fl)**	8.2 (7.5 – 8.7)^a^	7.6 (7.4 – 8.7)^a^	8.7 (8.4 – 9. 3)	<0.001*
**PCT (%)**	0.20 (0.18 – 0.26)^a^,^b^	0.18 (0.14 – 0.22)	0.19 (0.15 – 0.23)	<0.001*
**PDW (fl)**	13.3 (12.1 – 14.9)^a^,^b^	12.9 (11.2 – 14.2)^a^	14.5 (14.0 – 15.2)	<0.001*
**PLCR (%)**	13.1 (10.0 – 15.6)^a^	10.4 (7.0 – 18.0)^a^	16.8 (13.9 – 20.5)	0.001*
**D-dimer** **(ng/ml)**	244.6 (117.0 – 557.2)^a^,^b^	375.3 (129.8 – 833.5)^a^	116.6 (79.2 – 158.4)	<0.001*

Result reported as Median (IQR . Interquartile range)

* Median difference significant at p≤0.05

a median significantly different compared with control subject at p≤0.05

b median significantly different compared with surgical patients at p≤0.05

WBC - White blood cell, RBC - Red blood cell, HGB - Haemoglobin, HCT - Haematocrit, MCV - Mean cell volume, MCH - Mean cell haemoglobin, MCHC - Mean cell haemoglobin concentration, PLT - Platelet, MPV - Mean platelet volume, PCT - Plateletcrit, PDW - Platelet volume distribution width, PLCR - Platelet larger cell ratio

**Table 3:** Association of Haematological profile and D-dimer level with high risk of VTE occurrence in Medical patients

	Univariate Logistic Regression		Multivariate Logistic Regression	
Parameter	Odds Ratio (95% CI)	P-value	Adjusted Odds Ratio (95% CI)	P-value
**WBC (×10^9^/L)**	1.18 (1.04 – 1.34)	0.010*	1.27 (1.08 – 1.51)	0.005*
**HGB (g/dl)**	1.36 (1.15 – 1.61)	<0.001*	1.04 (0.83 – 1.30)	0.723
**PLT (×10^9^/L)**	1.00 (0.99 – 1.00)	0.700	1.01 (1.00 – 1.01)	0.002*
**MPV (fl)**	1.13 (0.87 – 1.46)	0.372	0.85 (0.60 – 1.21)	0.357
**PCT (%)**	12.14 (0.25–591.65)	0.208	5.69x104 (213.01–1.52x107)	<0.001*
**PDW (fl)**	1.71 (1.37 – 2.15)	<0.001*	1.70 (1.33 – 2.16)	<0.001*
**PLCR (%)**	1.09 (1.03 – 1.15)	0.003*	1.01 (0.94 – 1.09)	0.741
**D-dimer (ng/ml)**	1.00 (0.99 – 1.00)	<0.001*	1.00 (0.99 – 1.00)	0.006*

CI: Confidence interval, PDW:platelete volume distribution,PLCR: Platelet larger cell ratio, PLT - Platelet, PCT: Plateletcrit,, MPV: Mean platelet volume, WBC : White blood cell, HGB: Haemoglobin

**Table 4:** Association of Haematological profile and D-dimer level with high risk of VTE occurrence in Surgical patients

	Univariate Logistic Regression		Multivariate Logistic Regression	
Parameter	Odds Ratio (95% CI)	P-value	Adjusted Odds Ratio (95% CI)	P-value
**WBC (x10^9^/L)**	1.34 (1.08 – 1.66)	0.008*	38.08 (6.13 – 236.68)	<0.001*
**HGB (g/dl)**	0.59 (0.47 – 0.75)	<0.001*	0.43 (0.24 – 0.78)	0.006*
**PLT (x10^9^/L)**	1.00 (0.99 – 1.01)	0.176	1.01 (1.00 – 1.02)	0.037*
**MPV (fl)**	0.83 (0.58 – 1.19)	0.311	0.43 (0.20 – 0.92)	0.029*
**PCT (%)**	8.30 (0.05–1366.46)	0.416	2.3x109 (733.65–7.3x1015)	0.996
**PDW (fl)**	1.02 (0.87 – 1.20)	0.787	1.03 (0.79 – 1.35)	0.830
**PLCR (%)**	0.99 (0.95 – 1.03)	0.625	0.948 (0.88 – 1.02)	0.172
**D-dimer (ng/ml)**	1.00 (0.99 – 1.00)	0.999	1.00 (1.00 – 1.00)	0.033*

CI: Confidence interval, PDW:platelete volume distribution,PLCR: Platelet larger cell ratio, PLT – Platelet, PCT: Plateletcrit,, MPV :Mean platelet volume, WBC : White blood cell, HGB: Haemoglobin.
